# Factors Associated with the Need for, and the Impact of, Extracorporeal Membrane Oxygenation in Children with Congenital Heart Disease during Admissions for Cardiac Surgery

**DOI:** 10.3390/children4110101

**Published:** 2017-11-22

**Authors:** Salvatore Aiello, Rohit S. Loomba

**Affiliations:** 1 Department of Pediatrics, Chicago Medical School, 3333 North Green Bay Road, North Chicago, IL 60064, USA; 2 Cincinnati Children’s Hospital, Division of Pediatric Cardiology, 3333 Burnet Avenue, Cincinnati, OH 45229, USA; loomba.rohit@gmail.com

**Keywords:** pediatrics, children, extracorporeal membrane oxygenation, congenital heart disease, risk factors, mortality, life support

## Abstract

Introduction: This study aimed to determine factors associated with the need for extracorporeal membrane oxygenation (ECMO) in children with congenital heart disease (CHD) during admission for cardiac surgery (CS). A secondary aim was to determine how ECMO impacted length, cost, and mortality of the admission. Methods: Data from the Kids’ Inpatient Database (KIDS) were utilized. Admissions with CHD under 18 years of age with cardiac surgery were included. Need for ECMO in these admissions was then identified. Univariate analysis was conducted to compare characteristics between admissions with and without ECMO. Regression analyses were conducted to determine what factors were independently associated with ECMO and whether ECMO independently impacted admission characteristics. Results: A total of 46,176 admissions with CHD and CS were included in the final analysis. Of these, 798 (1.7%) required ECMO. Median age of ECMO admissions was 0.5 years. The following were associated with ECMO: decreased age, heart failure, acute kidney injury, arrhythmia, double outlet right ventricle, atrioventricular septal defect, transposition, Ebstein anomaly, hypoplastic left heart syndrome, common arterial trunk, tetralogy of Fallot, coronary anomaly, valvuloplasty, repair of total anomalous pulmonary venous connection, arterial switch, RV to PA conduit placement, and heart transplant (*p* < 0.01). ECMO independently increased length of stay by 17.8 days, cost of stay by approximately $415,917, and inpatient mortality 22-fold. Conclusion: Only a small proportion of CHD patients undergoing CS require ECMO, although these patients require increased resource utilization and have high mortality. Specific cardiac lesions, cardiac surgeries, and comorbidities are associated with increased need for ECMO.

## 1. Introduction

Each year, 1% of live births are diagnosed with a congenital heart defect (CHD) [1,2]. Since the first interventions, surgical correction has allowed for an increasing number of these patients to survive into adulthood, requiring care designed for life beyond the first year, and not just palliative care [2,3,4].

Over the past four years, there have been over 20,000 neonatal and 30,000 infant patients receiving corrective surgery for CHD, with mortality rates of 8.8% and 2.8% respectively [5,6]. This mortality has remained relatively stable over recent years, and attempts to further decrease this number now require a detailed understanding of the risk factors for mortality. One such risk factor of mortality in pediatric patients undergoing cardiac surgery is the need for extracorporeal membrane oxygenation (ECMO) [7].

Between 2–5% of pediatric CHD patients undergoing cardiac surgery will require ECMO [8]. While this is a small population, there has been an increase in CHD patients receiving ECMO despite these patients having the highest mortality rate among all pediatric patients receiving ECMO [9,10].

There is currently a paucity of data regarding risk factors that can help identify those who will require postoperative ECMO. We conducted an analysis of the Kids’ Inpatient Database (KIDS) with the primary aim of identifying the factors associated with increased risk of ECMO in pediatric patients undergoing cardiac surgery. Secondary aims were to quantify the effects of postoperative ECMO length of stay, cost of admission, and inpatient mortality.

## 2. Methods

### 2.1. Patient Identification

A retrospective cohort study was conducted using the pediatric database, KIDS. Data regarding hospital admissions was obtained from the 2005–2012 KIDS iterations. Only admissions of patients under 18 years of age with congenital heart disease undergoing heart surgery were included. These were identified using International Classifcation of Diseases (ICD-9) diagnosis and procedure codes as below.

Congenital heart disease was identified using ICD-9 codes: double outlet right ventricle using 745.11, atrioventricular septal defect using 745.60, partial anomalous pulmonary venous connection using 747.42, total anomalous pulmonary venous connection using 747.41, transposition of the great arteries using 745.10, congenitally corrected transposition using 745.12, hypoplastic left heart syndrome using 746.7, atrial septal defect using 745.61, ventricular septal defect using 745.5, pulmonary atresia with ventricular septal defect using 746.01, tricuspid atresia using 746.1, Ebstein anomaly using 746.2, truncus arteriosus using 745.0, and coronary artery anomaly using 746.85. Arrhythmias were identified using codes 427.0–427.42 as well as 426.0–426.13. It was not possible to distinguish between those in the absence of a spleen or multiple spleens due to the ICD-9 coding strategy.

Cardiac surgery was identified using the following codes: codes 35.10–35.14 for valvuloplasty with no valve replacement, 35.20–35.28 for valvuloplasty with replacement, codes 35.50–35.73 for septal defect repair (including atrioventricular septal defect repair), 35.81 for complete repair of tetralogy of Fallot, 35.82 for pulmonary venous repair, 35.83 for complete repair of common arterial trunk, 35.84 for arterial switch operation for transposition, 35.91 for atrial switch operation for transposition, 35.92 for right ventricle to pulmonary artery conduit, 39.22 for Blalock-Tausig shunt, and 37.51 for heart transplant.

ECMO was identified using ICD-9 code 39.65. It should be noted that timing of ECMO relative to the surgery could not be established by this database.

The following syndromes were also captured: Noonan, Turner, DiGeorge, trisomy 13, trisomy 18, trisomy 21, and syndrome not otherwise specified.

### 2.2. Data Identification and Collection

Demographic information including, age and race were collected for each admission. Comorbid conditions, as well as admission characteristics such as admission month, length of stay, and cost of stay, were collected and assessed. This research did not directly involve human participants, as this was previously collected data from a national registry. Informed consent was obtained by the national registry, but was not required by our specific study.

### 2.3. Statistical Analysis

Baseline characteristics such as age, race, and comorbid conditions were compared between those with and without ECMO (Table 1). Continuous variables are reported using median and range, while categorical variables are reported using absolute frequency and percentages. Continuous variables were analyzed using Student’s *t*-test or the Mann-Whitney-U test as appropriate, with categorical variables being analyzed using chi-square analysis.

Logistic regression was then conducted to determine factors associated with an increased or decreased need for ECMO, with ECMO as the dependent variable. 

Finally, to determine the effects of ECMO on outcomes, linear regression analyses was conducted with ECMO as the independent variable with length of stay, cost of admission, and mortality of ECMO admissions as dependent variables.

All statistical analysis was done utilizing SPSS Version 20.0 (IBM Corp, Chicago, IL, USA).

## 3. Results

### 3.1. Admission Characteristics in Those with and without ECMO (Univariate Analysis)

A total of 46,176 admissions with CHD and a documented cardiac surgery were included in the analysis. Of these, 798 (1.7%) required ECMO. It is not possible from this database to determine the timing of ECMO relative to cardiac surgery. Those requiring ECMO were significantly more likely to be younger (0.5 years versus 3.3 years, *p* < 0.01). Comorbidities most likely in those with ECMO included heart failure (odds ratio 2.2, 95% confidence interval (CI) = 1.9–2.6, *p* < 0.01), acute kidney injury (OR 20.1, 95%CI 17.0–23.8, *p* < 0.01), and arrhythmia (not including atrioventricular block) (OR 4.3, 95%CI 3.4–5.5, *p* < 0.01) (Table 1).

With regard to syndromes, Noonan syndrome was present in 2.3% of those requiring ECMO compared to 1.7% of those not requiring ECMO (*p* = 0.20). No patients with Turner syndrome were placed on ECMO. Down syndrome was present in 3.5% of those who were placed on ECMO compared to 10.7% of those not requiring ECMO (*p* < 0.01). DiGeorge syndrome was found in 4.5% of those who were placed on ECMO compared to 2.0% of those not placed on ECMO (*p* < 0.01). No patients with Trisomy 13 or 18 were placed on ECMO. A syndrome not otherwise specified was present in 2.3% of those placed on ECMO compared to 1.7% of those not requiring ECMO (*p* = 0.20).

Length of the admission was significantly longer in those with ECMO (49.2 days versus 12.5 days, *p* < 0.01), as was cost of the admission ($727,830 versus $141,524, *p* < 0.01). Inpatient mortality was also greater in those with ECMO, with 46.4% mortality compared to 1.8% in those without (OR 47.1, 95%CI 40.3–55.0, *p* < 0.01) (Table 1).

### 3.2. Cardiac Morphology and Surgery in Those with and without ECMO

The following cardiac malformations were more likely in those with ECMO: double outlet right ventricle (OR 1.6, 95%CI 1.3–1.2, *p* < 0.01), total anomalous pulmonary venous connection (OR 4.8, 95%CI 3.8–6.1, *p* < 0.01), coronary artery anomaly (OR 2.4, 95%CI 1.6–3.5, *p* < 0.01), atrial septal defect (OR 0.4, 95%CI 0.4–0.5, *p* < 0.01), ventricular septal defect (OR 0.6, 95%CI 0.5–0.8, *p* < 0.01), pulmonary atresia (OR 1.5, 95%CI 1.1–2.2, *p* = 0.01), Ebstein anomaly (OR 2.3, 95%CI 1.3–4.0, *p* < 0.01), hypoplastic left heart syndrome (OR 2.6, 95%CI 2.2–3.2, *p* < 0.01), transposition (OR 2.6, 95%CI 2.0–3.4, *p* < 0.01), and common arterial trunk (OR 3.1, 95%CI 2.1–4.2, *p* < 0.01) (Table 1).

The following cardiac surgeries were more likely in those with ECMO: valvuloplasty, no valve replacement (OR 1.3, 95%CI 1.1–1.7, *p* = 0.01), valvuloplasty, with valve replacement (OR 0.7, 95%CI 0.5–0.9, *p* = 0.02), septal defect repair (OR 0.3, 95%CI 0.2–0.4, *p* < 0.01), common arterial trunk, complete repair (OR 3.9, 95%CI 2.6–5.7, *p* < 0.01), total anomalous pulmonary venous connection repair (OR 5.2, 95%CI 4.2–6.5, *p* < 0.01), transposition repair, arterial switch (OR 3.8, 95%CI 2.7–5.3, *p* < 0.01), transposition, atrial switch (OR 0.5, 95%CI 0.1–2.0), right ventricle to pulmonary artery conduit (OR 3.1, 95%CI 2.5–4.0, *p* < 0.01), and heart transplant (OR 9.7, 95%CI 7.7–12.4) (Table 1).

### 3.3. Independent Risk Factors Associated with ECMO (Logistic Regression Analysis)

The following risk factors were associated with an increase in ECMO use: heart failure (BC 0.4, OR 1.4, 95%CI 1.2–1.7), acute kidney injury (BC 2.3, OR 10.8, 95%CI 8.8–13.3), arrhythmia (BC 1.2, OR 3.4, 95%CI 2.5–4.6), double outlet right ventricle (DORV) (BC 0.3, OR 1.3, 95%CI 1.1–1.9), Atrioventricular septal defect (AVSD) (BC 0.3 OR 1.3, 95%CI 1.1–1.8), coronary anomaly (BC 0.6 OR 1.8, 95%CI 1.1–2.9), Transposition (BC 0.7, OR 2.1, 95%CI 1.4–3.1), Ebstein (BC 0.7, OR 2.0, 95%CI 1.1–3.9), HLHS (BC 0.8, OR 2.2, 95%CI 1.6–2.9), HLHS (BC 0.8, OR 2.2, 95%CI 1.6–2.9), Common arterial trunk (BC 1.0, OR 2.8, 95%CI 1.4–5.4), Tetralogy of Fallot (BC 0.4, OR 1.5, 95%CI 1.1–2.3), Valvuloplasty no replacement (BC 1.4, OR 4.1, 95%CI 2.8–6.1), Valvuloplasty w/replacement (BC 1.3, OR 3.6, 95%CI 2.3–5.6), TAPVC complete repair (BC 2.0, OR 7.1, 95%CI 3.8–13.2), Arterial switch (BC 1.2, OR 3.4, 95%CI 1.9–6.1), right ventricle to pulmonary artery conduit (BC 1.4, OR 4.1, 95%CI 2.7–5.9), and heart transplant (BC 2.6, OR 13.9, 95%CI 9.2–21.2) (Table 2).

The following risk factors were associated with a decrease in ECMO use: Age (BC −0.2, OR 0.8, 95%CI 0.8–0.8) and Atrial septal defect (BC −0.3, OR 0.7, 95%CI 0.6–0.9).

### 3.4. Effect of ECMO on Admission Characteristics (Regression Analysis)

After multivariate analysis, the use of ECMO was associated with significant (*p* < 0.01) increases in the length, cost, and mortality of admissions. ECMO increased the length of admission by 17.8 days, increased the cost of admission by approximately $415,917, and increased inpatient mortality approximately 22.4-fold (Table 3).

## 4. Discussion

In our study, 1.7% of pediatric patients undergoing cardiac surgery required ECMO. This is lower than the 4% previously reported in the literature [8,11]. This may be due to an era effect as this percentage has tended to increase with time while the time period of our study lags by a few years [9]. The median age for patients requiring ECMO in our study was 0.5 years, with younger patients being more likely to require ECMO. This finding is consistent with other studies that have also demonstrated a greater need for ECMO in neonates and infants [9,12,13]. In patients receiving ECMO, the length of admission was nearly triple that for those who did not receive ECMO, a finding consistent with previous studies that have demonstrated that ECMO increases ICU and overall admission durations [12,14,15]. There was also a sharp increase in mortality from under 2% for non-ECMO to nearly 50% for EMCO patients; an unfortunate trend that has been noted in many centers [9,16].

ECMO is used in CHD patients suffering from a broad spectrum of malformations and abnormalities, requiring specific and unique care tailored to the management of the respective diseases [17,18,19,20,21]. Our study identifies specific risk factors associated with increased risk of ECMO. Identification of patients who may be at higher risk for ECMO may allow for postoperative care to be altered in specific patients or for increased vigilance in specific patients deemed to be at higher risk for ECMO. Ultimately, this may allow for decreased ECMO utilization and the subsequent morbidity and mortality [22].

Models to predict the need for ECMO and ICU length of stay do exist [14,23,24]. These models, however, are not intended specifically for pediatric CHD patients. The SAVE-score is a robust model that can aid in predicting need for ECMO, and subsequent survival, in adults. This scoring system even includes data regarding CHD. However, the model was not developed for pediatric patients and does not take into account the nuances of care in neonates and infants [25].

As discussed earlier, ECMO significantly increases cost of admission, in our study the median total cost of the admissions requiring ECMO was $727,830. Our study noted that ECMO independently increased the cost of admission by more than $400,000 [26]. Our study also demonstrated a median length of admission of 49 days for those requiring ECMO. This is consistent with a recent study that demonstrated that ECMO admissions were significantly more likely to require an ICU stay greater than 30 days [26].

While our study captured overall length of admission, we were unable to specifically identify the length of the ECMO run. In an analysis of the Pediatric Health Information System (PHIS) database from 2004–2013, Gupta and colleagues examined the relationship between the length of ECMO runs and outcomes in CHD patients after undergoing cardiac surgery [27]. ECMO runs under 7 days had no increase in mortality, but durations of ECMO longer than a week had significantly increased mortality, longer hospital stays, and increased hospital costs with every passing 24 h [27]. Our study has comparable findings, when considering our data versus that of Gupta and colleagues’ findings, irrespective of ECMO duration: mortality of 46.4% vs. 51.9%, median stay of 49.2 vs. 40 days, and median cost of $727,830 vs. $759,000. Our study was unable to capture length of ECMO run, and thus we cannot compare this to the data put forth by Gupta and colleagues.

The interplay between length and cost of admission is intuitive, but how both can be minimized in the context of admissions requiring ECMO is not clear. Some groups have suggested that one method by which to decrease the duration of ECMO runs, and overall admissions, may be cardiac catheterization. This can allow for the identification of residual lesions that can be intervened upon. Three studies have now demonstrated that this approach has led to improved survival and shortened ECMO runs [28,29,30].

Mortality rates of those afflicted with CHD have been decreasing over time [31]. This is due, in part, to the steadily increasing survival rate of pediatric cardiac ECMO cases. Despite improvements, however, overall mortality still remains as high as 51% [9]. These findings closely align with the 46.4% mortality noted for ECMO admissions in our study. While our study benefits from a large sample size, as well as from the number of specific conditions that were able to be examined, our study also has its limitations. We are limited in our knowledge of center-specific data, which precludes us from properly adjusting mortality rates to patient volumes at centers, which is known to impact patient mortalities [10]. Additionally, ICD-9 coding for congenital heart disease has not been rigorously evaluated yet, and thus some patients may not have been identified by the ICD-9 codes utilized. The temporal relationship of occurrences is also not explicit in the database, and thus a temporal assumption is being made.

Nonetheless, we feel that our study highlights factors associated with higher risk for ECMO, and delineates the future directions of such research. Identification of those at higher risk may allow for better understanding of how ECMO can be avoided in specific patients or how ECMO runs can be shortened.

## 5. Conclusions

A small proportion of pediatric patients will require ECMO during admission for cardiac surgery. Specific cardiac malformations and surgeries are independently associated with increased risk of ECMO. Those requiring ECMO have increased length and cost of stay. This study found inpatient mortality to be 46.4% in pediatric patients requiring ECMO during admission for cardiac surgery.

## Figures and Tables

**Table 1:** Characteristics of admissions with and without ECMO.

	No ECMO (*n* = 45,378)	ECMO (*n* = 798)	Odds Ratio (95%CI)	*p*-Value
**Age (years)**	3.3 (0–17)		--	<0.01
**Race**				0.357
White	19,544 (53.4)	343 (54.4)		
Black	4410 (12.0)	81 (12.9)		
Hispanic	8198 (22.4)	128 (20.3)		
Asian or Pacific Islander	1510 (4.1)	22 (3.5)		
Native American	296 (0.8)	*** (***)		
Other	2671 (7.3)	54 (8.6)		
**Heart failure**	8476 (18.7)	274 (34.3)	2.2 (1.9 to 2.6)	<0.01
**Acute kidney injury**	876 (1.9)	227 (28.4)	20.1 (17.0 to 23.8)	<0.01
**Arrhythmia (not including atrioventricular block)**	1070 (2.4)	76 (9.5)	4.3 (3.4 to 5.5)	<0.01
**Atrioventricular block**	894 (2.0)	14 (1.8)	0.8 (0.5 to 1.5)	0.66
**Cardiac lesion**				
Double outlet right ventricle	2428 (5.4)	69 (8.6)	1.6 (1.3 to 2.1)	<0.01
Atrioventricular septal defect	4679 (10.3)	100 (12.5)	1.2 (1.1 to 1.5)	0.04
Partial anomalous pulmonary venous connection	833 (1.8)	*** (***)	0.5 (0.2 to 1.1)	0.08
Total anomalous pulmonary venous connection	1063 (2.3)	83 (10.4)	4.8 (3.8 to 6.1)	<0.01
Coronary artery anomaly	695 (1.5)	29 (3.6)	2.4 (1.6 to 3.5)	<0.01
Atrial septal defect	18,560 (40.9)	204 (25.6)	0.4 (0.4 to 0.5)	<0.01
Tetralogy of Fallot	4721 (10.4)	83 (10.4)	1.0 (0.7 to 1.3)	0.99
Ventricular septal defect	13,367 (29.5)	177 (22.2)	0.6 (0.5 to 0.8)	<0.01
Pulmonary atresia	1140 (2.5)	31 (3.9)	1.5 (1.1 to 2.2)	0.01
Tricuspid atresia	1627 (3.6)	20 (2.5)	0.6 (0.4 to 1.1)	0.10
Ebstein anomaly	340 (0.7)	14 (1.8)	2.3 (1.3 to 4.0)	<0.01
Hypoplastic left heart syndrome	3337 (7.4)	139 (17.4)	2.6 (2.2 to 3.2)	<0.01
Transposition	1499 (3.3)	67 (8.4)	2.6 (2.0 to 3.4)	<0.01
Congenitally corrected transposition	323 (0.7)	*** (***)	1.2 (0.5 to 2.6)	0.58
Common arterial trunk	729 (1.6)	38 (4.8)	3.1 (2.1 to 4.2)	<0.01
**Cardiac surgery**				
Valvuloplasty, no valve replacement	3277 (7.2)	76 (9.5)	1.3 (1.1 to 1.7)	0.01
Valvuloplasty with valve replacement	3731 (8.2)	48 (6.0)	0.7 (0.5 to 0.9)	0.02
Septal defect repair	23,159 (51.0)	198 (24.8)	0.3 (0.2 to 0.4)	<0.01
Tetralogy of Fallot, complete repair	3332 (7.3)	51 (6.4)	0.8 (0.6 to 1.1)	0.30
Common arterial trunk, complete repair	433 (1.0)	29 (3.6)	3.9 (2.6 to 5.7)	<0.01
Total anomalous pulmonary venous connection repair	1145 (2.5)	96 (12.0)	5.2 (4.2 to 6.5)	<0.01
Transposition repair, arterial switch	585 (1.3)	38 (4.8)	3.8 (2.7 to 5.3)	<0.01
Transposition, atrial switch	223 (0.5)	*** (***)	0.5 (0.1 to 2.0)	0.33
Right ventricle to pulmonary artery conduit	1491 (3.3)	78 (9.8)	3.1 (2.5 to 4.0)	<0.01
Blalock-Tausig shunt	95 (0.2)	*** (***)	1.7 (0.5 to 5.6)	0.31
Heart transplant	560 (1.2)	87 (10.9)	9.7 (7.7 to 12.4)	<0.01
**Length of hospital stay (days)**	12.5 (1–22)	49.2 (1 to 145)	--	<0.01
**Cost of hospitalization (USD)**	141,524	727,830	--	<0.01
**Inpatient mortality**	817 (1.8)	370 (46.4)	47.1 (40.3 to 55.0)	<0.01

*** Represents an absolute frequency less than 10 which per database policies cannot be explicitly reported; CI—confidence interval.

**Table 2:** Risk factors for ECMO. Results of logistic regression analysis demonstrating independent factors significantly associated with increased and decreased risk of ECMO.

Factors Independently Associated with Need for ECMO	
Increase in ECMO	Decrease in ECMO
➢ Heart failure (BC 0.4, OR 1.4, 95%CI 1.2–1.7)	➢ Age (BC −0.2, OR 0.8, 95%CI 0.8–0.8)
➢ Acute kidney injury (BC 2.3, OR 10.8, 95%CI 8.8–13.3)	➢ Atrial septal defect (BC −0.3, OR 0.7, 95%CI 0.6–0.9)
➢ Arrhythmia (BC 1.2, OR 3.4, 95%CI 2.5–4.6)	
➢ DORV (BC 0.3, OR 1.3, 95%CI 1.1–1.9)	
➢ AVSD (BC 0.3, OR 1.3, 95%CI 1.1–1.8)	
➢ Coronary anomaly (BC 0.6, OR 1.8, 95%CI 1.1–2.9)	
➢ Transposition (BC 0.7, OR 2.1, 95%CI 1.4–3.1)	
➢ Ebstein (BC 0.7, OR 2.0, 95%CI 1.1–3.9)	
➢ HLHS (BC 0.8, OR 2.2, 95%CI 1.6–2.9)	
➢ Common arterial trunk (BC 1.0, OR 2.8, 95%CI 1.4–5.4)	
➢ Tetralogy of Fallot (BC 0.4, OR 1.5, 95%CI 1.1–2.3)	
➢ Valvuloplasty no replacement (BC 1.4, OR 4.1, 95%CI 2.8–6.1)	
➢ Valvuloplasty w/replacement (BC 1.3, OR 3.6, 95%CI 2.3–5.6)	
➢ TAPVC complete repair (BC 2.0, OR 7.1, 95%CI 3.8–13.2)	
➢ Arterial switch (BC 1.2, OR 3.4, 95%CI 1.9–6.1)	
➢ RV to PA conduit (BC 1.4, OR 4.1, 95%CI 2.7–5.9)	
➢ Heart transplant (BC 2.6, OR 13.9, 95%CI 9.2–21.2)	

BC—beta coefficient, OR—odds ratio, 95%CI—95% confidence interval, DORV—double outlet right ventricle, TAPVC—total anomalous pulmonary venous connection.

**Table 3:** Impact of ECMO on admission characteristics. Results of regression analyses demonstrating the independent impact of ECMO on length of stay, cost of stay, and mortality.

Effect of ECMO on Admission Characteristics				
	Beta-Coefficient	Odds Ratio	95%CI	*p*-Value
**Length of Stay**	17.8	--	--	<0.01
**Cost of Stay**	415,917	--	--	<0.01
**Mortality**	3.1	22.4	18.1–27.7	<0.01
